# Associations between the timing of different foods’ consumption with cardiovascular disease and all-cause mortality among adults with sleep disorders

**DOI:** 10.3389/fnut.2022.967996

**Published:** 2022-09-29

**Authors:** Jia Zhang, Yuntao Zhang, Lin Liu, Xuanyang Wang, Xiaoqing Xu, Ying Li, Tianshu Han, Wei Wei

**Affiliations:** National Key Discipline, Department of Nutrition and Food Hygiene, School of Public Health, Harbin Medical University, Harbin, China

**Keywords:** sleep disorder, chrono-nutrition, cardiovascular diseases, mortality, NHANES

## Abstract

**Introduction:**

People with sleep disorders are under disrupted biological rhythms. Whether changing the timing of specific food consumption contributes to decreasing cardiovascular and all-cause risk is unknown.

**Methods:**

A total of 8,005 participants with sleep disorders were selected from the U.S. National Health and Nutrition Examination Survey (NHANES) from 2005 to 2014. Cox proportional hazards regression models were used to analyze the relationship between the consumption time of foods and cardiovascular disease (CVD) and all-cause death. Moreover, equivalent food substitution models were carried out to evaluate the alterations in the risk of CVD mortality for the changed food intake time.

**Results:**

After adjusting for multiple confounders, participants who consume red and orange vegetables, starchy vegetables, and fermented dairy in the morning (hazard ratio (*HR*)_*red and orange vegetables*_ = 0.45, 95% CI: 0.26–0.81; *HR*_*starchy vegetables*_ = 0.47, 95% CI: 0.25–0.88; *HR*_*fermented dairy*_ = 0.57, 95% CI: 0.36–0.89) and milk and eggs in the evening contribute to reducing the likelihood of death from CVD (*HR*_*milk*_ = 0.65, 95% CI: 0.43–0.96; *HR*_*eggs*_ = 0.72, 95% CI: 0.53–0.98). Iso-calorically switching 0.1 serving of starchy vegetable and fermented dairy and milk intake from one period to another does significantly reduce the mortality risk of CVD.

**Conclusion:**

Higher intake of red and orange vegetables, starchy vegetables, and fermented dairy in the morning and milk and eggs in the evening confers a lower risk of CVD among individuals with sleep disorders.

## Introduction

Sleep disorders have become a new public health burden worldwide. Epidemiological surveys show that people with insufficient sleep alone account for one-third of the total adults in the USA (1, 2). Sleep disorder has been shown to be associated with multiple chronic diseases (3, 4), among which cardiovascular disease (CVD), as the leading cause of death worldwide, accounts for about one-third of global deaths (5, 6). The mechanism between sleep disorders and CVD could be explained by the relationships between sleep, circadian disruption, and metabolic physiology (7), which means that the disordered circadian rhythm of inflammation, oxidative stress, and sympathetic activity induced by sleep disorders further contribute to endothelial dysfunction and metabolic disturbances (8, 9). However, the circadian rhythm disorder caused by sleep disorders could be improved by accepted modifiable behaviors, among which adjusting the dietary factor is the most economical and convenient way (10–13).

However, most studies related to dietary interventions focus on the quantity or quality of dietary factors (14, 15). As an emerging field of nutritional research, chrono-nutrition aims to emphasize the importance of dietary intake time for health (16). It is well known that circadian rhythms could regulate a variety of physiological activities, such as food intake, which in turn feedback regulates nutrient absorption, distribution, metabolism, and excretion by driving peripheral circadian clocks (16). In short, the concept of “chrono-nutrition” is mainly derived from the abovementioned coordination between food intake time and the circadian body rhythm, which is broadly covers in three parts: (1) the distribution of energy intake in a day; (2) the frequency of food intake per day; and (3) the timing of food intake (17). Several studies published recently successively demonstrated the close association between the fields of chrono-nutrition and health (18–21). Circadian rhythms have a strong association between sleep and food intake time, but few studies investigated whether and how the timing of food consumption affects CVD in people with sleep disturbances. Therefore, our study was conducted to examine the association between the intake time of different foods and the mortality of all-cause and CVD among individuals with sleep disorders, aiming to provide practical intervention strategies for the prevention and treatment of CVD.

## Materials and methods

### Study population

The National Health and Nutrition Examination Survey (NHANES) is a stratified, multistage study conducted by the U.S. National Center for Health Statistics (NCHS). The researcher used professional interviews and examinations to collect nationally representative data of American civilians. Adults with a sleep duration of less than 6 h, self-reporting sleep trouble, or diagnosed with a sleep disorder by a doctor-were classified as having a sleep disorder (22, 23). After excluding the participants who had missed dietary intake and/or mortality variables, a total of 8,005 participants with sleep disorders (3,636 men and 4,370 women) were selected. The NHANES data obtained the approval of the NCHS Institutional Review Board and the informed consent of the participants and could be available through https://wwwn.cdc.gov/nchs/nhanes/Default.aspx.

### Dietary and sleep duration assessment

The dietary food intake of each participant was assessed over 2 non-consecutive days through 24-h diet recalls. Dietary foods and energy intake were estimated based on the guidelines of the U.S. Department of Agriculture’s Dietary Research Food and Nutrition Database. According to the user guide of the MPED2.0 of the U.S. Department of Agriculture Survey Foods, 15 different food groups were selected and used for further analysis. A sleep questionnaire was used to assess the sleep status of each participant. Based on the time point of food intake, these meals are classified as breakfast, lunch, dinner, and snacks.

### Main exposure

The food groups included in this study were mainly whole grains, refined grains, dark green vegetables, red and orange vegetables, starchy vegetables, total fruit, milk, fermented dairy, eggs, red meat, poultry, cured meat, seafood, soybean products, and legumes. The exposure variables were set as food intakes in the morning, afternoon, and evening.

### Main outcome

The main outcome variable was the mortality of CVD and all causes, which was determined by the National Death Index (NDI). As a highly reliable resource, NDI is widely used for death identification. The International Classification of Diseases 10th Revision (ICD-10) is used to determine disease-specific death, among which ICD-10 codes, such as I00–I09, I11, I13, I20–I51, or I60–I69 are defined as CVD mortality. A total of 658 deaths, including 189 deaths due to CVD, were used for further analysis.

### Confounding and effect modification measurements

Potential covariates are as follows: age (years), sex (men/women), race/ethnicity (non-Hispanic white/non-Hispanic black/Mexican American/other), annual household income level (less than $20,000, between $20,000 and $45,000, between $45,000 and $75,000, between $75,000 and $100,000, or over $100,000), educational level (below 9th grade, between 9th and 11th grade, finish high school, finish GED or equivalent, finish college or Associate in Arts degree, or finish college graduate or above), regular exercise (yes/no), cigarette and alcohol use (yes/no), body mass index (BMI) (kg/m^2^), disease history of diabetes, hypertension, and dyslipidemia (yes/no), covered by health insurance (yes/no), total energy intake (kcal/day), total fat intake (g/day), total carbohydrate intake (g/day), total protein intake (g/day), whole grains (ounce equivalents), refined grains (ounce equivalents), dark green vegetables (cup equivalents), red and orange vegetables (cup equivalents), starchy vegetables (cup equivalents), fruit (cup equivalents), milk (cup equivalents), fermented dairy (cup equivalents), eggs (ounce equivalents), red meat (ounce equivalents), poultry (ounce equivalents), cured meat (ounce equivalents), seafood (ounce equivalents), soybean products (ounce equivalents), legumes (ounce equivalents), and the Alternative Healthy Eating Index (AHEI).

### Statistical analysis

The intake of food groups in the morning, afternoon, and evening were divided into two (whether or not to eat) or three (base on the distribution) parts according to their distribution. For social demographics, lifestyle and eating behavior, and anthropometric indicators, categorical variables were represented by percentages, and continuous variables were represented by median (P25, P75). The baseline characteristics were compared by the Mann–Whitney *U*-test and the Chi-square test. R 4.0.2 was used to conduct all statistical analyses, and a two-sided *p*-value < 0.05 was considered to be statistically significant. The median was used to replace missing values for covariates with less than 5% missing values.

Cox proportional hazards models were established to evaluate the relationship between food intake across a day and CVD and all-cause mortality. Confounding factors were adjusted in all models, such as age, gender, race, BMI, drinking, smoking, exercise, income, education, total energy intake, total fat intake, total carbohydrate intake, total protein intake, AHEI, health insurance coverage, disease history of diabetes, hypertension, and dyslipidemia. In addition, the total intake of every food group in a 24-h period was adjusted in the model.

An equivalent food substitution model was carried out to evaluate the alterations in the risk of CVD mortality for the changed food intake time. A substitution analysis is mainly conducted by converting food intake time points and keeping the total energy and other food intake constant.

### Sensitivity analysis

In set 1, sensitivity analyses were performed to assess the relationship between total red and orange vegetable, starchy vegetable, milk, fermented dairy, and egg intake in a whole day and CVD and all-cause mortality to check whether the intake time could provide more information. In set 2, sensitivity analyses were performed among individuals with sound sleep to evaluate the impact of sleep status on the results. In set 3, AHEI was additionally adjusted to evaluate the impact of dietary quality on the results. In set 4, a sensitivity analysis was conducted to evaluate whether sex, diabetes, hypertension, and dyslipidemia could affect the relationship between food intake time and CVD and all-cause risk.

## Results

### Baseline characteristics

The demographic and nutrition characteristics on the basis of the disease status of CVD mortality are presented in Table 1. Compared with other participants, the participants who died due to CVD were more likely to be men, less likely to be non-Hispanic white, had a higher age, the prevalence of dyslipidemia, hypertension, and diabetes, and a lower percentage of health insurance coverage, exercise, education, income, total energy, fat, protein, carbohydrate intake, and AHEI (*P* < 0.05).

**TABLE 1:** The baseline characteristics of studying variables by disease status.

Variables	CVD mortality (*N* = 189)	Non-CVD mortality (*N* = 7,817)	*P*-value
Age (years)	73.00 (64.00, 80.00)	50.00 (37.00, 63.00)	<0.001
Men (%)	116 (61.37)	3,250 (45.03)	0.995
Non-Hispanic white (%)	16 (8.46)	873 (11.16)	0.002
Current smoking (%)	46 (24.33)	2,350 (30.06)	0.233
Current drinking (%)	129 (68.25)	5,677 (72.62)	0.184
Regular exercise (%)	12 (6.34)	1,255 (16.05)	0.001
College graduate or above (%)	20 (10.58)	1,570 (20.08)	<0.001
>$100,000 annual household income (%)	7 (3.70)	924 (11.82)	<0.001
BMI (kg/m^2^)	28.75 (24.70, 33.02)	29.00 (24.94, 34.06)	0.295
Total energy intake (kcal/day)	1,610.50 (1,222.50, 2,005.00)	1,884.00 (1,431.50, 2,460.50)	<0.001
Total fat intake (g/day)	58.57 (39.64, 81.69)	70.00 (49.28, 96.69)	<0.001
Total protein intake (g/day)	64.01 (48.01, 86.38)	72.39 (53.95, 96.04)	<0.001
Total carbohydrate intake (g/day)	202.05 (155.91, 252.87)	229.23 (171.50, 301.96)	<0.001
AHEI	47.00 (37.00, 55.00)	52.00 (42.00, 62.00)	<0.001
Diabetes (%)	63 (33.33)	1,513 (19.36)	<0.001
Hypertension (%)	157 (83.07)	3,830 (49.00)	<0.001
Dyslipidemia (%)	98 (51.85)	3,059 (39.13)	<0.001
Covered by health insurance (%)	6,383 (81.7)	180 (95.2)	<0.001
Whole grain (ounce equivalents)	0.55 (0.00, 1.24)	0.42 (0.00, 1.18)	0.159
Refined grain (ounce equivalents)	4.01 (2.71, 5.91)	4.75 (3.09, 6.87)	0.001
Dark green vegetable (cup equivalents)	0.00 (0.00, 0.00)	0.00 (0.00, 0.11)	0.012
Red and orange vegetable (cup equivalents)	0.37 (0.06, 0.84)	0.51 (0.19, 1.02)	<0.001
Starchy vegetable (cup equivalents)	0.66 (0.00, 1.41)	0.55 (0.00, 1.27)	0.632
Fruit (cup equivalents)	0.89 (0.25, 1.43)	0.63 (0.05, 1.41)	0.005
Milk (cup equivalents)	0.62 (0.17, 1.20)	0.45 (0.12, 1.05)	0.010
Fermented dairy (cup equivalents)	0.47 (0.09, 1.00)	0.28 (0.00, 0.67)	<0.001
Eggs (ounce equivalents)	0.28 (0.03, 0.89)	0.22 (0.03, 0.88)	0.357
Red meat (ounce equivalents)	0.90 (0.00, 2.23)	1.00 (0.00, 2.38)	0.178
Poultry (ounce equivalents)	0.41 (0.00, 2.09)	0.83 (0.00, 2.26)	0.050
Cured meat (ounce equivalents)	4.97 (2.96, 7.65)	4.17 (2.66, 7.18)	0.039
Seafood (ounce equivalents)	0.00 (0.00, 0.28)	0.00 (0.00, 0.43)	0.454
Soybean products (ounce equivalents)	0.00 (0.00, 0.00)	0.00 (0.00, 0.00)	0.018
Legumes (ounce equivalents)	0.00 (0.00, 0.00)	0.00 (0.00, 0.40)	0.021

Continuous variables are presented as median (P25, P75). Categorical variables are presented as percentages. Hypertension is defined by a self-reported diagnosis, systolic blood pressure >140 mmHg and/or diastolic blood pressure >90 mmHg. Hyperlipidemia is defined as serum triglyceride ≥2.26 mmol/L, or serum cholesterol ≥6.22 mmol/L, or low-density lipoprotein ≥4.14 mmol/L. Diabetes is defined by a self-reported diagnosis, an HbA1c level ≥6.5%, or a fasting plasma glucose level ≥7.0 mmol/L. AHEI, Alternative Healthy Eating Index.

### Associations of foods intake time with all-cause and cardiovascular disease mortality

The association of red and orange vegetable, starchy vegetable, milk, fermented dairy, and egg intake consumed in the morning and evening with all-cause and CVD mortality is presented in Table 2 among participants with sleep disorders. In addition, the association of other dietary foods consumed in the morning and evening with CVD and all-cause mortality is presented in Supplementary Table 1. The results indicated that participants who eat red and orange vegetables, starchy vegetables, and fermented dairy in the morning had lower CVD mortality, compared with participants who skip red and orange vegetables, starchy vegetables, and fermented dairy in the morning, as indicated by hazard ratio (*HR*) and 95% CI (*HR*_red and orange vegetables_ = 0.46, 95% CI: 0.26–0.82; *HR*_starchy vegetables_ = 0.47, 95% CI: 0.25–0.88; and *HR*_fermented dairy_ = 0.57, 95% CI: 0.36–0.90). Furthermore, compared with participants in the highest quintile of milk intake in the evening, those in the lowest quintile were more likely to die due to CVD, as indicated by *HR* and 95% CI (*HR*_*milk*_ = 0.65, 95% CI: 0.43–0.96). Similarly, the consumption of eggs in the evening also confers a lower risk of CVD (*HR*_eggs_ = 0.72, 95% CI: 0.53–0.98). Moreover, participants who consumed red and orange vegetables in the morning and fermented dairy in the evening had a lower association with all-cause mortality (*HR*_red and orange vegetables_ = 0.75, 95% CI: 0.58–0.97; and *HR*_fermented dairy_ = 0.77, 95% CI: 0.64–0.92).

**TABLE 2:** Multivariate adjusted hazard ratios (*HR*s) of the dietary red and orange vegetables, starchy vegetables, milk, fermented dairy, and eggs intake in the morning and the evening and CVD-mortality and all-cause mortality among participants with sleep disorders.

	CVD mortality		All-cause mortality	
	Case/*N*	HR (95% CI)	Case/*N*	HR (95% CI)
**In the morning**				
**Red and orange vegetables (yes/no)**				
No	176/6,838	1	588/6,838	1
Yes	13/1,168	0.46 (0.26, 0.82)	70/1,168	0.75 (0.58, 0.97)
*P* for trend		0.008		0.030
**Starchy vegetables (yes/no)**				
No	178/7,099	1	595/7,099	1
Yes	11/907	0.47 (0.25, 0.88)	63/907	0.82 (0.62, 1.07)
*P* for trend		0.017		0.143
**Milk (tertiles)**				
Q1	51/2,401	1	180/2,401	1
Q2	59/2,721	0.87 (0.59, 1.28)	218/2,721	0.92 (0.75, 1.13)
Q3	79/2,884	0.75 (0.48, 1.18)	260/2,884	0.79 (0.63, 1.01)
*P* for trend		0.279		0.063
**Fermented dairy (yes/no)**				
No	166/5,966	1	547/5,966	1
Yes	23/2,040	0.57 (0.36, 0.90)	111/2,040	0.82 (0.66, 1.02)
*P* for trend		0.015		0.074
**Eggs (yes/no)**				
No	97/4,338	1	346/4,338	1
Yes	92/3,668	0.93 (0.66, 1.31)	312/3,668	0.99 (0.82, 1.18)
*P* for trend		0.667		0.869
In the evening				
**Red and orange vegetables (yes/no)**				
No	68/5,131	1	225/2,079	1
Yes	121/3,999	0.83 (0.59, 1.17)	433/5,927	0.84 (0.70, 1.01)
*P* for trend		0.281		0.062
**Starchy vegetables (yes/no)**				
No	92/2,079	1	225/2,079	1
Yes	97/5,927	0.74 (0.51, 1.07)	433/5,927	0.93 (0.76, 1.14)
*P* for trend		0.109		0.483
**Milk (tertiles)**				
Q1	56/2,470	1	173/2,470	1
Q2	63/2,309	1.07 (0.74, 1.54)	179/2,309	1.01 (0.82, 1.25)
Q3	70/3,227	0.65 (0.43, 0.96)	306/3,227	1.03 (0.83, 1.27)
*P* for trend		0.006		0.835
**Fermented dairy (yes/no)**				
No	108/3,397	1	371/3,397	1
Yes	81/4,609	0.77 (0.55, 1.08)	287/4,609	0.77 (0.64, 0.92)
*P* for trend		0.131		0.004
Eggs (yes/no)				
No	83/3,296	1	266/3,296	1
Yes	106/4,710	0.72 (0.53, 0.98)	392/4,710	0.91 (0.77, 1.07)
*P* for trend		0.039		0.256

Adjustments included age, gender, race, body mass index (BMI), drinking, smoking, exercise, income, education, total energy intake, total fat intake, total carbohydrate intake, total protein intake, AHEI, covered by health insurance, disease history of diabetes, disease history of hypertension, disease history of dyslipidemia, and total intake of specific food group in the 24-h period. Case/*N*, number of case participants/total; Q, quarter; AHEI, Alternative Healthy Eating Index.

### Equivalent substitution analysis

**FIGURE 1:**
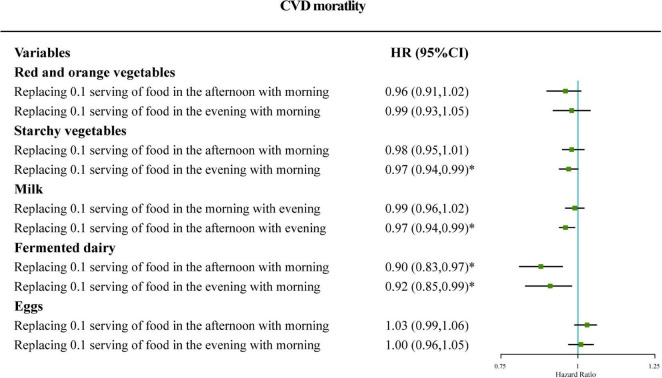
Adjusted hazard ratio (HR) for cardiovascular disease (CVD) mortality: iso-caloric substitution of red and orange vegetables, starchy vegetables, and fermented dairy consumed in the afternoon or the evening to morning and iso-caloric substitution of milk and eggs in the morning or the afternoon to the evening. Adjustments included age, gender, race, BMI, drinking, smoking, exercise, income, education, total energy intake, total fat intake, total carbohydrate intake, total protein intake, AHEI, health insurance coverage, disease history of diabetes, disease history of hypertension, disease history of dyslipidemia, and total intake of specific food group in the 24 h period. Case/*N*, number of case participants/total; Q, quarter; AHEI, Alternative Healthy Eating Index.

Figure 1 shows that the mortality risk of CVD in the predicted equivalent substitution models through switching food intake from one period to another among participants with sleep disorders. Figure 1 shows that the *HR* of CVD mortality decreased by 3% (*HR* = 0.97, 95% CI: 0.94–0.99) in models with 0.1 serving of starchy vegetables in the evening being equivalently switched to morning. Similarly, the results indicated that *HR*s for CVD decreased by 12 or 9% (*HR* = 0.90, 95% CI: 0.83–0.97; and *HR* = 0.92, 95% CI: 0.85–0.99) in models with 0.1 serving of fermented dairy in the afternoon or evening being equivalently switched to the morning. In addition, it can be concluded that *HR*s for CVD decreased by 4% (*HR* = 0.97, 95% CI: 0.94–0.99) in models with 0.1 serving of milk intake in the afternoon being equivalently switched to the evening.

### Sensitivity analysis

The result of the first sensitivity analysis showed that the total intake of red and orange vegetables, starchy vegetables, milk, fermented dairy, and eggs was not associated with CVD and all-cause mortality, which suggested that the analysis of the dietary intake time could provide more information than the total daily food intake only (Supplementary Table 2). The second set of the sensitivity analysis demonstrated that the association between intake of fermented dairy in the morning and milk and eggs in the evening with CVD mortality disappeared among participants with normal sleep (Supplementary Table 3), which means that the intake time of the above foods may play a role in reducing CVD death by improving the adverse effects of sleep or sleep deprivation among participants with sleep disorders. The third set of the sensitivity analysis indicated that, after additionally adjusting dietary quality, the relationship between the consumption time of the above foods and CVD was still significant (Supplementary Table 4). The fourth set of the sensitivity analysis showed that sex, diabetes, hypertension, and dyslipidemia could not affect the relationship between the consumption time of the above food items and CVD (Supplementary Tables 5–7).

## Discussion

Our study showed that people with sleep disorders who have red and orange vegetables, starchy vegetables, and fermented dairy consumption in the morning and milk and eggs in the evening seem to have decreased CVD mortality. In addition, while keeping food quality and quantity constant, switching 0.1 serving of milk intake from afternoon to morning, switching 0.1 serving of fermented dairy intake from afternoon or evening to morning, or switching 0.1 serving of starchy vegetables intake from evening to morning confer a lower risk of CVD.

Most studies focused on investigating the association between quantity and quality of different foods or dietary patterns and CVD (14, 15). However, as we all know, this was the first study to explore the association of the consumption time of dietary foods with CVD mortality among individuals with sleep disorders. Abundant studies illustrated that sleep disorders could activate inflammatory gene expression and the production of proinflammatory cytokines in the morning by increasing sympathetic nerve activity at midnight, which gradually decreases under normal circumstances, and exacerbate impairment on vascular reactivity in the morning (24–27). Based on the above evidence, attenuating the inflammatory response may be a way for reducing adverse effects caused by sleep disorders in the morning. As a type of food rich in anti-inflammatory phytochemicals, such as lycopene and carotenoids, red orange vegetables have been found to reduce oxidative stress and inflammation by influencing inflammatory markers and their downstream targets (28–30). This indicated that the benefits of a higher intake of red orange vegetables in the morning may be explained by the anti-inflammatory effects of phytochemicals, which suggests that we should pay more attention to increase its intake in the morning rather than other foods in the case of sleep disorder.

In addition to attenuating inflammatory effects, the interaction of sleep disorders and metabolic disorders is a basis of CVD (31, 32), which should be paid more attention. First, sleep disorders have been shown to increase the total amount and time of energy intake (33), which manifests as a decrease in energy intake at breakfast and an increase in energy intake at dinner, with fat and carbohydrate accounting for a large proportion of energy source at dinner (34, 35). However, evidence indicated that carbohydrate metabolism have an internal biological rhythm, which is highest in the morning and gradually decreases in the evening (36). It means that the right time of carbohydrates consumption should be in the morning rather than in the evening, which is the same as our result. In addition, another study demonstrated that higher energy intake at breakfast with lower intake at dinner have beneficial effects on metabolic control (37). Similarly, several random control trails showed that the carbohydrate-rich breakfast and fermented dairy could decrease food intake after lunch or later by increasing satiety and decreasing hunger rating (38–40), which supports the beneficial effect of high intakes of starchy vegetables and fermented dairy in the morning. The mechanism of the benefit of eating eggs in the evening in our results is still opaque. As a food source of high-quality protein, eggs have been shown to increase satiety and decrease food intake later (41). In addition, consuming eggs in the evening has been proven to improve glycometabolism, compared with other high-carbohydrate protein-matched sources (42). It demonstrated that eggs may be a suitable energy source in the evening and may help to improve metabolic disorders by reducing excessive energy intake in the evening.

Studies showed that most hormones regulate metabolism and energy balance through own circadian rhythms (43). Circadian rhythm disturbance caused by a sleep disorder has a great impact on the role of hormones. First of all, under normal circumstances, insulin sensitivity, and insulin secretion will decrease at night and increase in the morning (44). Therefore, people with insulin rhythm disturbances caused by sleep disorders should adapt to their normal rhythmic activity at an appropriate time, which is consistent with our result that starchy vegetables should be eaten in the morning (45). In addition, the nocturnal secretion of the appetite-related hormone leptin is reduced due to sleep disorders, which leads to an increase in total energy intake to energy imbalance (46). Consistent with the above statement, the energy imbalance is mainly manifested in the increased intake of high-carbohydrate and high-fat foods at night (35). However, as a decomposition hormone, cortisol itself has a low secretion level at night (44), and the dual effects of increased nighttime energy intake and decreased catabolism lead to increased susceptibility to metabolic disturbances. Therefore, it is recommended to consume some satiety foods, such as eggs at night to restrict energy intake. Melatonin, an important hormone that maintains the biological clock and regulates body rhythms (47), is also affected by sleep disorders, resulting in decreased secretion at night (44). Therefore, drinking milk rich in tryptophan at night helps to provide precursors for the synthesis of melatonin and improves sleep.

In addition to attenuating inflammatory effects and metabolic disorders, improving sleep may be another way to affect cardiovascular health. Our results found that the intake of milk and eggs in the evening helps to reduce CVD mortality. As the precursor of serotonin and melatonin, milk-rich tryptophan has been shown to play a major role in sleep/wake and cortical activity and contributes to promoting sleep (48–50). These studies provided the potential mechanism and evidence for the association of higher milk consumption in the evening and lower mortality of CVD in this study. Moreover, taking egg protein hydrolyzate before sleep also has been shown to improve sleep quality (51, 52), which may be another reason to reduce adverse effects of sleep disorder after eating eggs in the evening.

Moreover, the association between foods consumption time and CVD mortality was improved but not statistically protective after switching red and orange vegetable and egg intake time points, which may also be explained by lower energy intake in the morning caused by the excessive single red and orange vegetable intake and the high energy intake in the evening caused by excessive egg intake in the food substitution analysis. Therefore, it is suggested that we should consider the dietary quality, quantity, and time of food intake in all direction so as to avoid the nutritional imbalance caused by excessive attention to one part, which results in adverse consequences.

Based on our results above, we put forward the following suggestions for people with sleep disorders on the basis of keeping energy distribution balanced and reasonable in all three meals. Carbohydrates are best eaten in the forenoon, and in the selection of high-carbohydrate foods types, it is highly recommended to choose high-fiber-carbohydrate foods, such as starchy vegetables rather than high-energy carbohydrate-type foods, such as refined grains. High fiber-carbohydrate foods combined with red and orange vegetables rich in lycopene and carotenoids for breakfast provide energy in a nutritionally balanced manner and are more beneficial to combat the increased levels of oxidative stress and inflammation caused by sleep disorders. Fermented dairy products should be selected for forenoon snacks, which not only have higher nutritional value but also have a satiety effect to reduce subsequent energy intake. For dinner, a moderate amount of high-quality protein meals, such as eggs, should be used to replenish energy and increase satiety after meals. While reducing the adverse effects of excessive energy intake on sleep, a moderate intake of tryptophan-rich milk can also help improve sleep.

The advantages and limitations of our study are presented in the following. First, it is the first time to explore the relationship between the consumption time of foods and CVD mortality among individuals with sleep disorders. Then, consistent results were observed after adjusting lots of important confounding factors and conducting multiple sensitivity analyses in the process of research. Besides, the NHANES provides the nationally representative data in the USA, such as dietary intake and lifestyle factors. In addition, our research has certain limitations. First, the dietary data were only obtained through the 24-h diet recall on 2 days within 2 weeks, which cannot reflect long-term exposure. Second, the status of sleep condition was assessed by a single sleep disorder questionnaire, which is highly subjective and less accurate, compared with a real-time sleep monitoring equipment. In addition, other factors related to sleep disorders that might not be aware of or measured can also bring about bias.

## Conclusion

There are some important implications in our findings. People with sleep disorders are under a disrupted biological rhythm, which indicates that ill-rested people should pay more attention to eating habits due to their worse health condition. Accumulating evidence has suggested that food consumption time is no less important than quantity and quality for maintaining health. Based on the above results, we believe that the specific food consumption time play an important role in changing the risk of CVD death among individuals with sleep disorders. Therefore, adjusting the intake time of different food groups is of importance in the prevention and treatment of CVD and should be integrated into the nutritional recommendation.

## Data availability statement

Publicly available datasets were analyzed in this study. This data can be found here: https://wwwn.cdc.gov/nchs/nhanes/Default.aspx.

## Ethics statement

The studies involving human participants were reviewed and approved by the NCHS Institutional Review Board. The patients/participants provided their written informed consent to participate in this study.

## Authors contributions

YL, TH, and WW designed the research and had primary responsibility for final manuscript. JZ and YZ performed all statistical analyses and wrote the manuscript. LL, XW, and XX conducted the data review. All authors contributed to conduct literature research and providing corresponding suggestion, and read and approved the final manuscript.
