# Supplementary material for: Associations between the timing of different foods’ consumption with cardiovascular disease and all-cause mortality among adults with sleep disorders
Source: Front Nutr. 2022 Sep 29;9:967996. doi: 10.3389/fnut.2022.967996 (PMC9560773; doi:10.3389/fnut.2022.967996)
Supplement: Supplementary file 1 [file Data_Sheet_1.docx]

Supplementary table 1. Adjusted HRs for whole grains, refined grains, dark green vegetables, total fruit, red meat, cured meat, poultry, seafood, soybean products, legumes in the morning and evening and CVD and all-cause mortality among individuals with sleep disorder.

|  | CVD mortality | | All-cause mortality | |
| --- | --- | --- | --- | --- |
|  | Case/N | HR (95%CI) | Case/N | HR (95%CI) |
| **In the morning** |  |  |  |  |
| Whole grains (Yes/No) | |  |  |  |
| No | 88/4394 | 1 | 342/4394 | 1 |
| Yes | 101/3612 | 1.09(0.80,1.49) | 316/3612 | 0.91(0.77,1.08) |
| *P* for trend |  | 0.584 |  | 0.273 |
| Refined grains (Tertiles) | |  |  |  |
| T1 | 44/2518 | 1 | 142/2158 | 1 |
| T2 | 84/3019 | 0.92(0.63,1.33) | 297/3019 | 1.11(0.91,1.36) |
| T3 | 61/2829 | 0.82(0.53,1.26) | 219/2829 | 1.01(0.81,1.28) |
| *P* for trend |  | 0.440 |  | 0.888 |
| Dark green vegetables (Yes/No) | |  |  |  |
| No | 188/7819 | 1 | 651/7819 | 1 |
| Yes | 1/187 | 0.20(0.02,1.74) | 7/187 | 0.50(0.21,1.18) |
| *P* for trend |  | 0.144 |  | 0.115 |
| Total fruit (Tertiles) | |  |  |  |
| T1 | 67/3641 | 1 | 260/3641 | 1 |
| T2 | 27/1187 | 1.02(0.64,1.60) | 97/1187 | 0.97(0.76,1.23) |
| T3 | 95/3178 | 1.18(0.80,1.73) | 301/3178 | 1.11(0.90,1.36) |
| *P* for trend |  | 0.381 |  | 0.250 |
| Red meat (Yes/No) | |  |  |  |
| No | 180/7528 | 1 | 2255/6477 | 1 |
| Yes | 9/478 | 0.73(0.37,1.47) | 2669/6597 | 0.71(0.48,1.04) |
| *P* for trend |  | 0.381 |  | 0.080 |
| Cured meat (Yes/No) | |  |  |  |
| No | 131/5186 | 1 | 429/5186 | 1 |
| Yes | 58/2820 | 0.77(0.56,1.07) | 229/2820 | 0.98(0.83,1.15) |
| *P* for trend |  | 0.120 |  | 0.776 |
| Poultry (Yes/No) | |  |  |  |
| No | 184/7608 | 1 | 633/7608 | 1 |
| Yes | 5/398 | 0.72(0.29,1.79) | 25/398 | 1.00(0.66,1.51) |
| *P* for trend |  | 0.478 |  | 0.959 |
| Seafood (Yes/No) | |  |  |  |
| No | 186/7854 | 1 | 646/7854 | 1 |
| Yes | 3/152 | 0.85(0.24,3.08) | 12/152 | 0.94(0.48,1.84) |
| *P* for trend |  | 0.807 |  | 0.862 |
| Soybean products (Yes/No) | |  |  |  |
| No | 184/7629 | 1 | 637/7629 | 1 |
| Yes | 5/377 | 1.65(0.49,5.58) | 21/377 | 1.26(0.66,2.41) |
| *P* for trend |  | 0.419 |  | 0.482 |
| legumes (Yes/No) | |  |  |  |
| No | 184/7696 | 1 | 638/7696 | 1 |
| Yes | 5/310 | 1.24(0.45,3.42) | 20/310 | 1.00(0.59,1.70) |
| *P* for trend |  | 0.672 |  | 0.998 |
| **In the evening** |  |  |  |  |
| Whole grains (Yes/No) | |  |  |  |
| No | 123/5497 | 1 | 456/5497 | 1 |
| Yes | 66/2509 | 1.02(0.74,1.39) | 202/2509 | 0.91(0.76,1.08) |
| *P* for trend |  | 0.928 |  | 0.259 |
| Refined grains (Tertiles) | |  |  |  |
| T1 | 71/2357 | 1 | 231/2357 | 1 |
| T2 | 76/3136 | 0.89(0.64,1.25) | 266/3136 | 0.94(0.78,1.13) |
| T3 | 41/2513 | 0.82(0.51,1.33) | 161/2513 | 0.94(0.73,1.21) |
| *P* for trend |  | 0.416 |  | 0.645 |
| Dark green vegetables (Yes/No) | |  |  |  |
| No | 162/6201 | 1 | 556/6201 | 1 |
| Yes | 27/1805 | 0.43(0.14,1.29) | 102/1805 | 0.59(0.34,1,01) |
| *P* for trend |  | 0.132 |  | 0.054 |
| Total fruit (Tertiles) | |  |  |  |
| T1 | 77/3923 | 1 | 298/3923 | 1 |
| T2 | 20/858 | 1.19(0.72,1.96) | 60/858 | 0.91(0.69,1.20) |
| T3 | 92/3225 | 1.10(0.78,1.55) | 300/3225 | 1.02(0.85,1.23) |
| *P* for trend |  | 0.731 |  | 0.667 |
| Red meat (Yes/No) | |  |  |  |
| No | 100/3661 | 1 | 323/3661 | 1 |
| Yes | 89/4345 | 0.71(0.49,1.04) | 335/4345 | 0.85(0.70,1.04) |
| *P* for trend |  | 0.080 |  | 0.111 |
| Cured meat (Yes/No) | |  |  |  |
| No | 18/774 | 1 | 72/774 | 1 |
| Yes | 171/7232 | 1.37(0.82,2.28) | 586/7232 | 1.14(0.88,1.47) |
| *P* for trend |  | 0.226 |  | 0.315 |
| Poultry (Yes/No) | |  |  |  |
| No | 112/4461 | 1 | 381/4461 | 1 |
| Yes | 77/3545 | 1.10(0.71,1.71) | 277/3545 | 1.16(0.92,1.47) |
| *P* for trend |  | 0.678 |  | 0.207 |
| Seafood (Yes/No) | |  |  |  |
| No | 159/6514 | 1 | 561/6514 | 1 |
| Yes | 30/1492 | 1.49(0.70,3.17) | 97/1492 | 1.14(0.73,1.78) |
| *P* for trend |  | 0.302 |  | 0.558 |
| Soybean products (Yes/No) | |  |  |  |
| No | 182/7476 | 1 | 624/7476 | 1 |
| Yes | 7/530 | 1.25(0.42,3.74) | 34/530 | 1.24(0.67,2.28) |
| *P* for trend |  | 0.696 |  | 0.497 |
| legumes (Yes/No) | |  |  |  |
| No | 161/6555 | 1 | 551/6555 | 1 |
| Yes | 28/1451 | 1.62(0.68,3.85) | 107/1451 | 1.06(0.67,1.69) |
| *P* for trend |  | 0.274 |  | 0.801 |

Adjustments included age, gender, race, BMI, drinking, smoking, exercise, income, education, total energy intake, total fat intake, total carbohydrate intake, total protein intake, covered by health insurance, disease history of diabetes, disease history of hypertension, disease history of dyslipidemia and total intake of specific food group. T, Tertile. HR, hazard ratio.

Supplementary table 2. Adjusted HRs for total red and orange vegetables, starchy vegetables, milk, fermented dairy and eggs intake in 24-hours period and CVD and all-cause mortality among individuals with sleep disorder.

|  | CVD mortality | | All-cause mortality | |
| --- | --- | --- | --- | --- |
|  | Case/N | HR (95%CI) | Case/N | HR (95%CI) |
| Red and orange vegetables (Tertiles) | |  |  |  |
| T1 | 60/1756 | 1 | 191/1756 | 1 |
| T2 | 77/3485 | 0.82(0.58,1.15) | 284/3485 | 0.91(0.75,1.09) |
| T3 | 52/2765 | 0.69(0.47,1.01) | 183/2765 | 0.73(0.59,0.90) |
| *P* for trend |  | 0.110 |  | 0.005 |
| Starchy vegetables (Tertiles) | |  |  |  |
| T1 | 57/2237 | 1 | 174/2237 | 1 |
| T2 | 51/2652 | 0.69(0.47,1.00) | 211/2652 | 0.94(0.77,1.16) |
| T3 | 81/3117 | 1.00(0.70,1.41) | 273/3117 | 1.10(0.90,1.33) |
| *P* for trend |  | 0.411 |  | 0.157 |
| Milk (Tertiles) | |  |  |  |
| T1 | 44/2245 | 1 | 147/2245 | 1 |
| T2 | 71/3229 | 0.92(0.63,1.35) | 263/3229 | 1.05(0.85,1.29) |
| T3 | 74/2532 | 1.25(0.84,1.86) | 248/2532 | 1.30(1.04,1.61) |
| *P* for trend |  | 0.140 |  | 0.008 |
| Fermented dairy (Tertiles) | |  |  |  |
| T1 | 87/2675 | 1 | 309/2675 | 1 |
| T2 | 68/2666 | 1.13(0.82,1.57) | 205/2666 | 0.91(0.76,1.09) |
| T3 | 34/2665 | 1.00(0.65,1.52) | 144/2665 | 1.02(0.82,1.26) |
| *P* for trend |  | 0.894 |  | 0.895 |
| Eggs (Tertiles) | |  |  |  |
| T1 | 45/1784 | 1 | 144/1784 | 1 |
| T2 | 71/3418 | 0.77(0.53,1.13) | 271/3418 | 0.93(0.76,1.14) |
| T3 | 73/2804 | 0.95(0.65,1.41) | 243/2804 | 1.01(0.81,1.25) |
| *P* for trend |  | 0.439 |  | 0.490 |

Adjustments included age, gender, race, BMI, drinking, smoking, exercise, income, education, total energy intake, total fat intake, total carbohydrate intake, total protein intake, covered by health insurance, disease history of diabetes, disease history of hypertension, disease history of dyslipidemia and total intake of specific food group. T, Tertile. HR, hazard ratio.

Supplementary table 3. Adjusted HRs for red and orange vegetables, starchy vegetables, milk, fermented dairy and eggs in the morning and evening and CVD and all-cause mortality among individuals with well-sleep.

|  | CVD mortality | | All-cause mortality | |
| --- | --- | --- | --- | --- |
|  | Case/N | HR (95%CI) | Case/N | HR (95%CI) |
| **In the morning** |  |  |  |  |
| Red and orange vegetables (Yes/No) | |  |  |  |
| No | 407/18333 | 1 | 1351/18333 | 1 |
| Yes | 49/3763 | 0.72(0.53,0.99) | 199/3763 | 0.85(0.73,1.00) |
| *P* for trend |  | 0.040 |  | 0.044 |
| Starchy vegetables (Yes/No) | |  |  |  |
| No | 425/19528 | 1 | 1413/19528 | 1 |
| Yes | 31/2568 | 0.63(0.43,0.92) | 137/2568 | 0.79(0.66,0.95) |
| *P* for trend |  | 0.016 |  | 0.011 |
| Milk (Tertiles) | |  |  |  |
| T1 | 108/6254 | 1 | 368/6254 | 1 |
| T2 | 134/7018 | 0.90(0.69,1.16) | 482/7018 | 0.97(0.84,1.11) |
| T3 | 214/8824 | 0.94(0.70,1.25) | 700/8824 | 0.90(0.77,1.06) |
| *P* for trend |  | 0.922 |  | 0.203 |
| Fermented dairy (Yes/No) | |  |  |  |
| No | 388/16453 | 1 | 1280/16453 | 1 |
| Yes | 68/5643 | 0.80(0.61,1.05) | 270/5643 | 0.90(0.79,1.04) |
| *P* for trend |  | 0.115 |  | 0.179 |
| Eggs (Yes/No) | |  |  |  |
| No | 246/11753 | 1 | 809/11753 | 1 |
| Yes | 210/10343 | 0.90(0.72,1.11) | 741/10343 | 1.01(0.90,1.13) |
| *P* for trend |  | 0.311 |  | 0.876 |
| **In the evening** |  |  |  |  |
| Red and orange vegetables (Yes/No) | |  |  |  |
| No | 153/5618 | 1 | 495/5618 | 1 |
| Yes | 303/16478 | 0.83(0.67,1.03) | 1055/16478 | 0.86(0.77,0.97) |
| *P* for trend |  | 0.082 |  | 0.016 |
| Starchy vegetables (Yes/No) | |  |  |  |
| No | 207/10596 | 1 | 663/10596 | 1 |
| Yes | 249/11500 | 0.97(0.76,1.24) | 887/11500 | 1.02(0.89,1.17) |
| *P* for trend |  | 0.795 |  | 0.787 |
| Milk (Tertiles) | |  |  |  |
| T1 | 125/7041 | 1 | 418/7041 | 1 |
| T2 | 107/5117 | 1.03(0.79,1.34) | 323/5117 | 0.94(0.81,1.09) |
| T3 | 224/9938 | 0.95(0.74,1.21) | 809/9938 | 1.03(0.90,1.18) |
| *P* for trend |  | 0.499 |  | 0.359 |
| Fermented dairy (Yes/No) | |  |  |  |
| No | 265/9560 | 1 | 859/9560 | 1 |
| Yes | 191/12536 | 0.87(0.70,1.08) | 691/12536 | 0.90(0.80,1.01) |
| *P* for trend |  | 0.198 |  | 0.068 |
| Eggs (Yes/No) |  |  |  |  |
| No | 191/9275 | 1 | 622/9275 | 1 |
| Yes | 265/12812 | 0.85(0.69,1.04) | 928/12812 | 0.95(0.85,1.07) |
| *P* for trend |  | 0.107 |  | 0.402 |

Adjustments included age, gender, race, BMI, drinking, smoking, exercise, income, education, total energy intake, total fat intake, total carbohydrate intake, total protein intake, covered by health insurance, disease history of diabetes, disease history of hypertension, disease history of dyslipidemia and total intake of specific food group. T, Tertile. HR, hazard ratio.

Supplementary table 4. Adjusted HRs for red and orange vegetables, starchy vegetables, milk, fermented dairy and eggs in the morning and evening and CVD and all-cause mortality after additionally adjusting dietary quality.

|  | CVD mortality | | All-cause mortality | |
| --- | --- | --- | --- | --- |
|  | Case/N | HR (95%CI) | Case/N | HR (95%CI) |
| **In the morning** |  |  |  |  |
| Red and orange vegetables (Yes/No) | |  |  |  |
| No | 176/6838 | 1 | 588/6838 | 1 |
| Yes | 13/1168 | 0.46(0.26,0.82) | 70/1168 | 0.76(0.58,0.98) |
| *P* for trend |  | 0.008 |  | 0.032 |
| Starchy vegetables (Yes/No) | |  |  |  |
| No | 178/7099 | 1 | 595/7099 | 1 |
| Yes | 11/907 | 0.47(0.25,0.88) | 63/907 | 0.82(0.63,1.08) |
| *P* for trend |  | 0.019 |  | 0.152 |
| Milk (Tertiles) | |  |  |  |
| T1 | 51/2401 | 1 | 180/2401 | 1 |
| T2 | 59/2721 | 0.88(0.59,1.29) | 218/2721 | 0.93(0.76,1.14) |
| T3 | 79/2884 | 0.76(0.48,1.18) | 260/2884 | 0.80(0.63,1.01) |
| *P* for trend |  | 0.272 |  | 0.064 |
| Fermented dairy (Yes/No) | |  |  |  |
| No | 166/5966 | 1 | 547/5966 | 1 |
| Yes | 23/2040 | 0.57(0.36,0.90) | 111/2040 | 0.83(0.67,1.03) |
| *P* for trend |  | 0.016 |  | 0.086 |
| Eggs (Yes/No) | |  |  |  |
| No | 97/4338 | 1 | 346/4338 | 1 |
| Yes | 92/3668 | 0.94(0.66,1.33) | 312/3668 | 0.99(0.82,1.19) |
| *P* for trend |  | 0.719 |  | 0.911 |
| **In the evening** |  |  |  |  |
| Red and orange vegetables (Yes/No) | |  |  |  |
| No | 68/5131 | 1 | 225/2079 | 1 |
| Yes | 121/3999 | 0.84(0.59,1.18) | 433/5927 | 0.84(0.70,1.01) |
| *P* for trend |  | 0.306 |  | 0.068 |
| Starchy vegetables (Yes/No) | |  |  |  |
| No | 92/2079 | 1 | 225/2079 | 1 |
| Yes | 97/5927 | 0.74(0.51,1.08) | 433/5927 | 0.96(0.78,1.17) |
| *P* for trend |  | 0.742 |  | 0.668 |
| Milk (Tertiles) | |  |  |  |
| T1 | 56/2470 | 1 | 173/2470 | 1 |
| T2 | 63/2309 | 1.07(0.74,1.55) | 179/2309 | 1.01(0.82,1.25) |
| T3 | 70/3227 | 0.64(0.43,0.96) | 306/3227 | 1.03(0.83,1.27) |
| *P* for trend |  | 0.006 |  | 0.843 |
| Fermented dairy (Yes/No) | |  |  |  |
| No | 108/3397 | 1 | 371/3397 | 1 |
| Yes | 81/4609 | 0.77(0.55,1.08) | 287/4609 | 0.77(0.64,0.92) |
| *P* for trend |  | 0.130 |  | 0.004 |
| Eggs (Yes/No) |  |  |  |  |
| No | 83/3296 | 1 | 266/3296 | 1 |
| Yes | 106/4710 | 0.73(0.54,0.99) | 392/4710 | 0.91(0.77,1.07) |
| *P* for trend |  | 0.042 |  | 0.267 |

Adjustments included age, gender, race, BMI, drinking, smoking, exercise, income, education, total energy intake, total fat intake, total carbohydrate intake, total protein intake, covered by health insurance, disease history of diabetes, disease history of hypertension, disease history of dyslipidemia and total intake of specific food group. T, Tertile. HR, hazard ratio.

Supplementary table 5. Modification effect of sex on the association of red and orange vegetables, starchy vegetables, milk, fermented dairy and eggs intake in the morning and evening with CVD and all-cause mortality.

| Intake time | Dietary food | CVD mortality | | All-cause mortality | |
| --- | --- | --- | --- | --- | --- |
|  |  | HR (95% CI) | *P* | HR (95% CI) | *P* |
| Morning | Red and orange vegetables | 0.41(0.05,3.47) | 0.410 | 0.77(0.39,1.52) | 0.453 |
|  | Starchy vegetables | 0.10(0.00,2.61) | 0.167 | 1.07(0.68,1.71) | 0762 |
|  | Milk | 0.92(0.50,1.70) | 0.789 | 0.87(0.63,1.19) | 0.375 |
|  | Fermented dairy | 0.64(0.25,1.64) | 0.349 | 0.62(0.40,0.95) | 0.027 |
|  | Eggs | 0.63(0.32,1.22) | 0.168 | 0.81(0.60,1.09) | 0.162 |
| Evening | Red and orange vegetables | 0.94(0.48,1.84) | 0.854 | 0.78(0.55,1.10) | 0.158 |
|  | Starchy vegetables | 1.08(0.79,1.47) | 0.631 | 1.01(0.84,1.20) | 0.948 |
|  | Milk | 0.60(0.30,1.20) | 0.146 | 1.08(0.81,1.43) | 0.602 |
|  | Fermented dairy | 1.76(0.97,3.21) | 0.064 | 1.46(1.07,2.00) | 0.018 |
|  | Eggs | 0.23(0.05,1.12) | 0.069 | 1.00(0.62,1.63) | 0.987 |

Adjustments included age, gender, race, BMI, drinking, smoking, exercise, income, education, total energy intake, total fat intake, total carbohydrate intake, total protein intake, covered by health insurance, disease history of diabetes, disease history of hypertension, disease history of dyslipidemia and total intake of specific food group. T, Tertile. HR, hazard ratio.

Supplementary table 6. Modification effect of diabetes, hypertension and dyslipidemia history on the association of red and orange vegetables, starchy vegetables, milk, fermented dairy and eggs intake in the morning and evening with CVD mortality.

| Intake time | Dietary food | Diabetes | | Hypertension | | Dyslipidemia | |
| --- | --- | --- | --- | --- | --- | --- | --- |
|  |  | HR (95% CI) | *P* | HR (95% CI) | *P* | HR (95% CI) | *P* |
| Morning | Red and orange vegetables | 0.00(0.00,858.46) | 0.152 | 1.38(0.16,12.31) | 0.771 | 0.52(0.10,2.57) | 0.418 |
|  | Starchy vegetables | 1.17(0.48,2.85) | 0.728 | 1.12(0.32,3.91) | 0.855 | 1.85(0.62,5.49) | 0.269 |
|  | Milk | 0.78(0.43,1.40) | 0.405 | 4.20(1.29,13.61) | 0.017 | 0.93(0.55,1.59) | 0.792 |
|  | Fermented dairy | 0.63(0.24,1.68) | 0.352 | 0.44(0.16,1.17) | 0.098 | 0.87(0.36,2.09) | 0.747 |
|  | Eggs | 0.89(0.55,1.44) | 0.630 | 0.99(0.58,1.69) | 0.957 | 1.10(0.71,1.71) | 0.659 |
| Evening | Red and orange vegetables | 1.12(0.58,2.14) | 0.742 | 1.10(0.47,2.57) | 0.826 | 0.91(0.49,1.69) | 0.767 |
|  | Starchy vegetables | 0.82(0.59,1.13) | 0.225 | 1.20(0.76,1.89) | 0.443 | 0.81(0.61,1.07) | 0.137 |
|  | Milk | 0.65(0.33,1.25) | 0.196 | 1.46(0.66,3.26) | 0.350 | 0.70(0.41,1.17) | 0.173 |
|  | Fermented dairy | 1.24(0.67,2.29) | 0.499 | 1.63(0.73,3.61) | 0.231 | 0.59(0.33,1.05) | 0.073 |
|  | Eggs | 1.26(0.54,2.93) | 0.600 | 3.26(0.42,25.45) | 0.261 | 0.80(0.35,1.86) | 0.609 |

Adjustments included age, gender, race, BMI, drinking, smoking, exercise, income, education, total energy intake, total fat intake, total carbohydrate intake, total protein intake, covered by health insurance, disease history of diabetes, disease history of hypertension, disease history of dyslipidemia and total intake of specific food group. T, Tertile. HR, hazard ratio.

Supplementary table 7. Modification effect of diabetes, hypertension and dyslipidemia history on the association of red and orange vegetables, starchy vegetables, milk, fermented dairy and eggs intake in the morning and evening with all-cause mortality.

| Intake time | Dietary food | Diabetes | | Hypertension | | Dyslipidemia | |
| --- | --- | --- | --- | --- | --- | --- | --- |
|  |  | HR (95% CI) | *P* | HR (95% CI) | *P* | HR (95% CI) | *P* |
| Morning | Red and orange vegetables | 0.78(0.40,1.50) | 0.453 | 1.54(0.69,3.44) | 0.297 | 0.96(0.53,1.73) | 0.891 |
|  | Starchy vegetables | 1.20(0.82,1.77) | 0.349 | 1.24(0.75,2.05) | 0.398 | 1.24(0.84,1.85) | 0.284 |
|  | Milk | 0.84(0.61,1.15) | 0.271 | 1.07(0.77,1.49) | 0.695 | 0.72(0.54,0.97) | 0.031 |
|  | Fermented dairy | 0.89(0.57,1.38) | 0.607 | 0.77(0.49,1.20) | 0.251 | 1.16(0.77,1.74) | 0.491 |
|  | Eggs | 1.02(0.79,1.30) | 0.903 | 1.00(0.78,1.28) | 0.990 | 1.18(0.93,1.48) | 0.168 |
| Evening | Red and orange vegetables | 1.21(0.88,1.68) | 0.245 | 1.14(0.78,1.65) | 0.504 | 0.89(0.65,1.22) | 0.454 |
|  | Starchy vegetables | 0.87(0.72,1.05) | 0.140 | 0.94(0.79,1.13) | 0.524 | 0.91(0.78,1.07) | 0.249 |
|  | Milk | 0.68(0.49,0.95) | 0.025 | 0.93(0.70,1.23) | 0.605 | 0.71(0.54,0.93) | 0.011 |
|  | Fermented dairy | 1.23(0.88,1.70) | 0.224 | 1.18(0.83,1.69) | 0.364 | 0.95(0.70,1.30) | 0.759 |
|  | Eggs | 1.02(0.65,1.61) | 0.937 | 0.96(0.58,1.60) | 0.880 | 0.93(0.60,1.45) | 0.756 |

Adjustments included age, gender, race, BMI, drinking, smoking, exercise, income, education, total energy intake, total fat intake, total carbohydrate intake, total protein intake, covered by health insurance, disease history of diabetes, disease history of hypertension, disease history of dyslipidemia and total intake of specific food group. T, Tertile. HR, hazard ratio.
